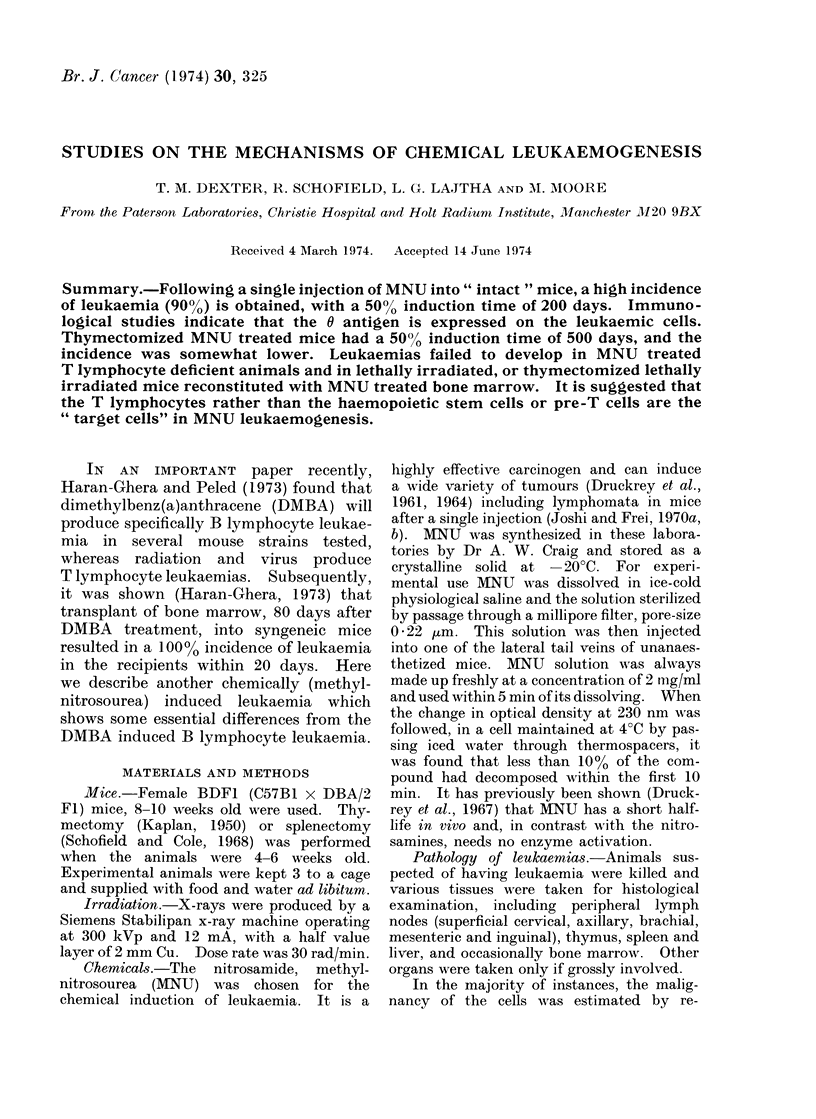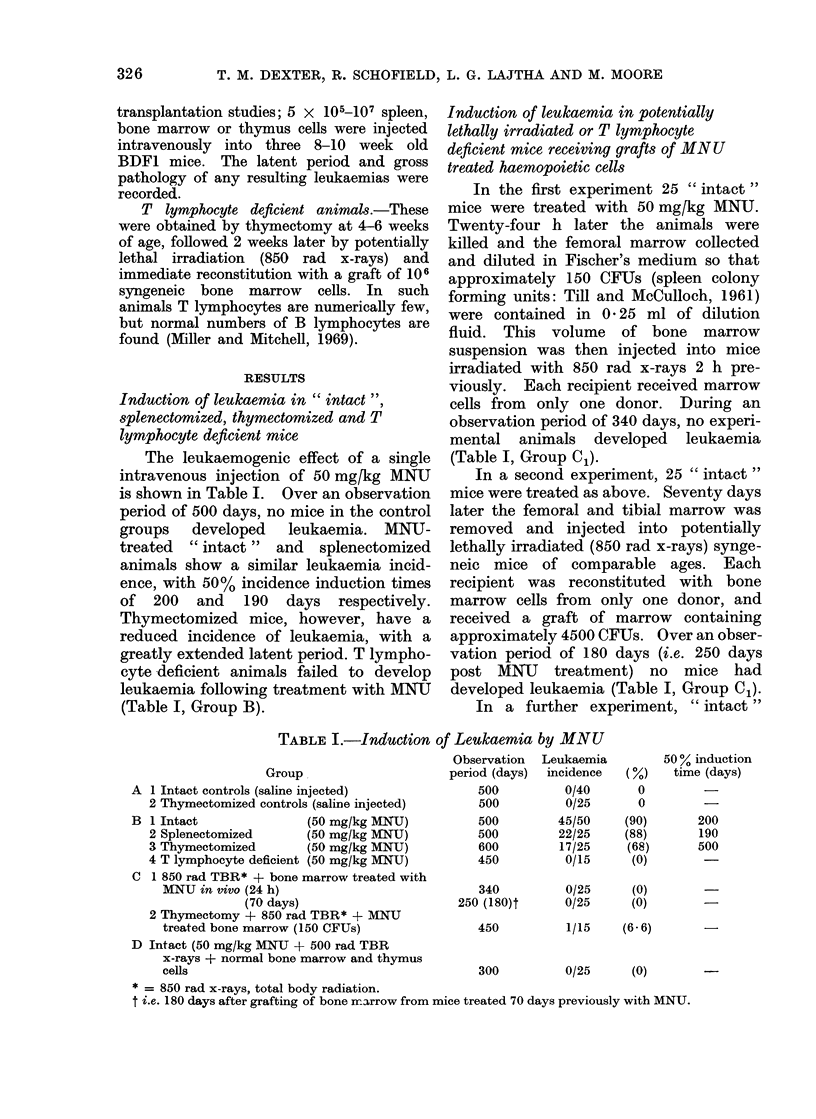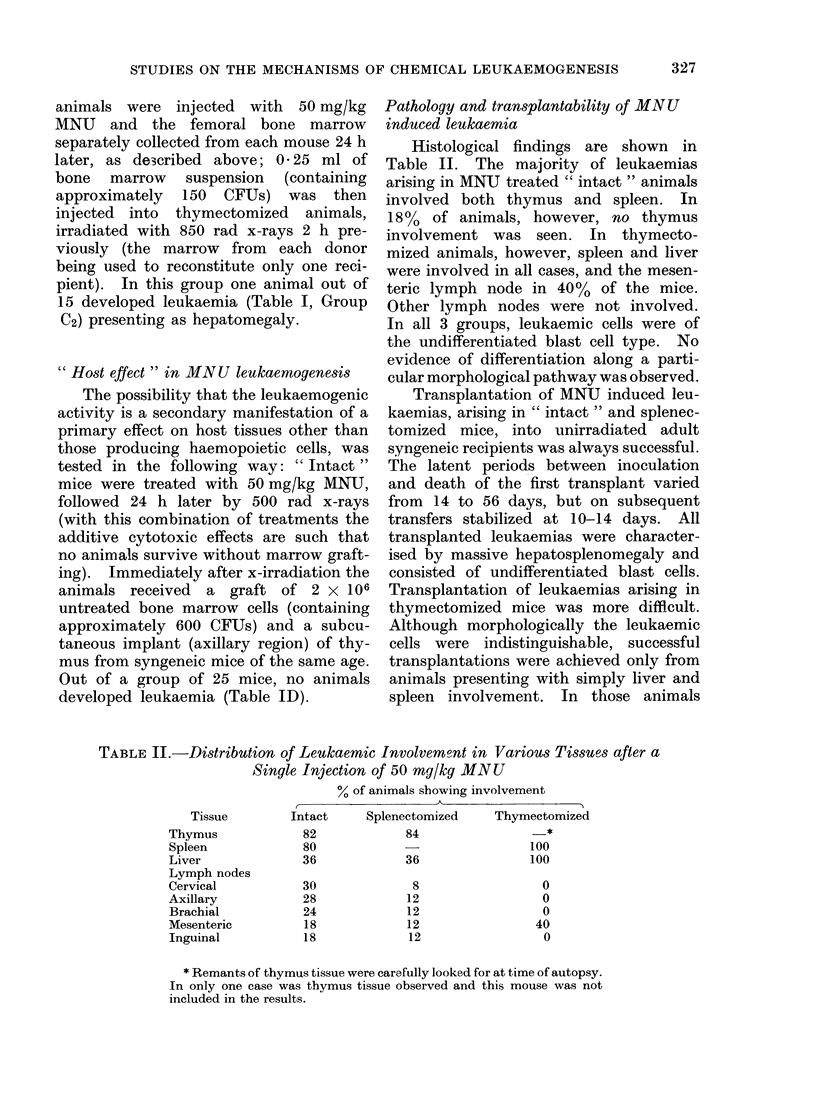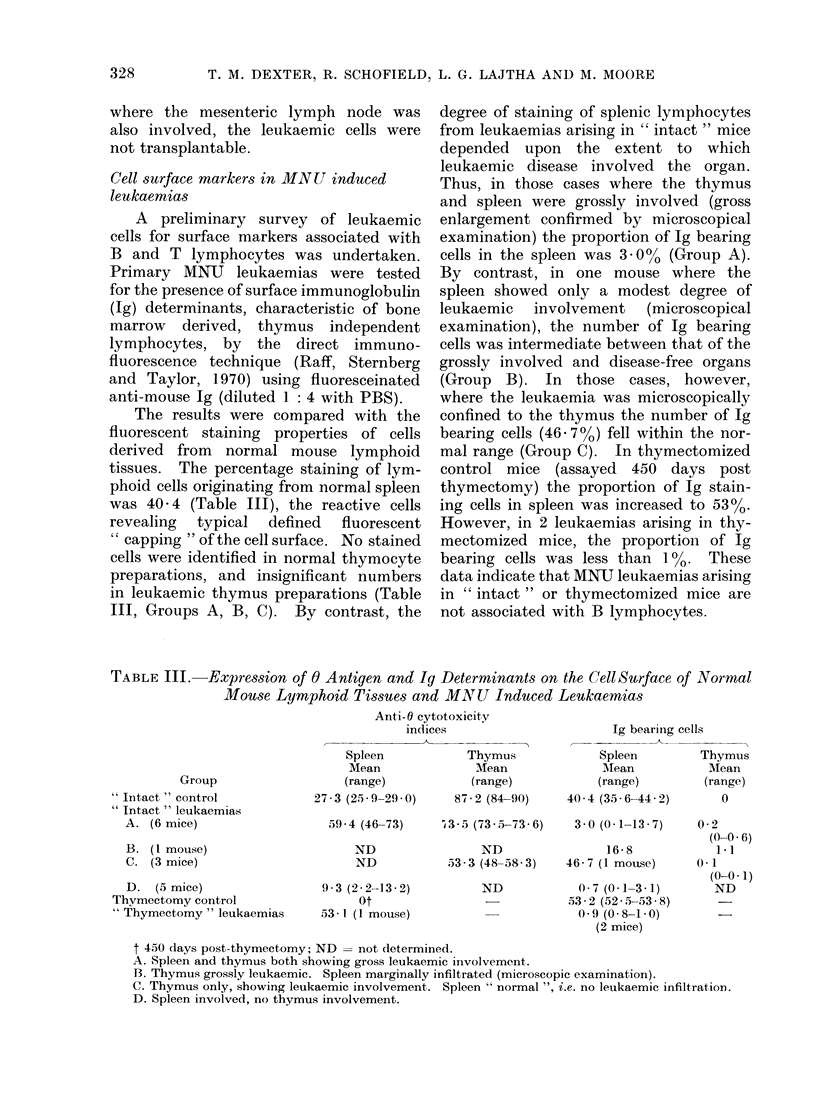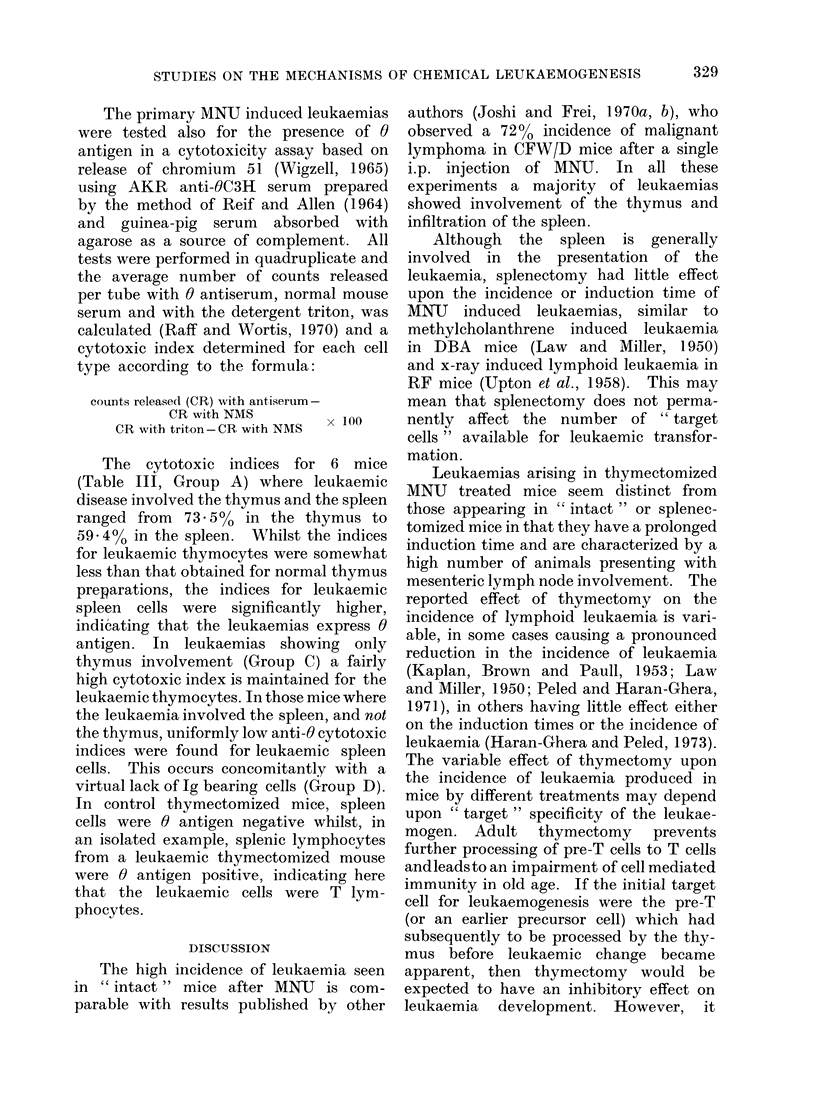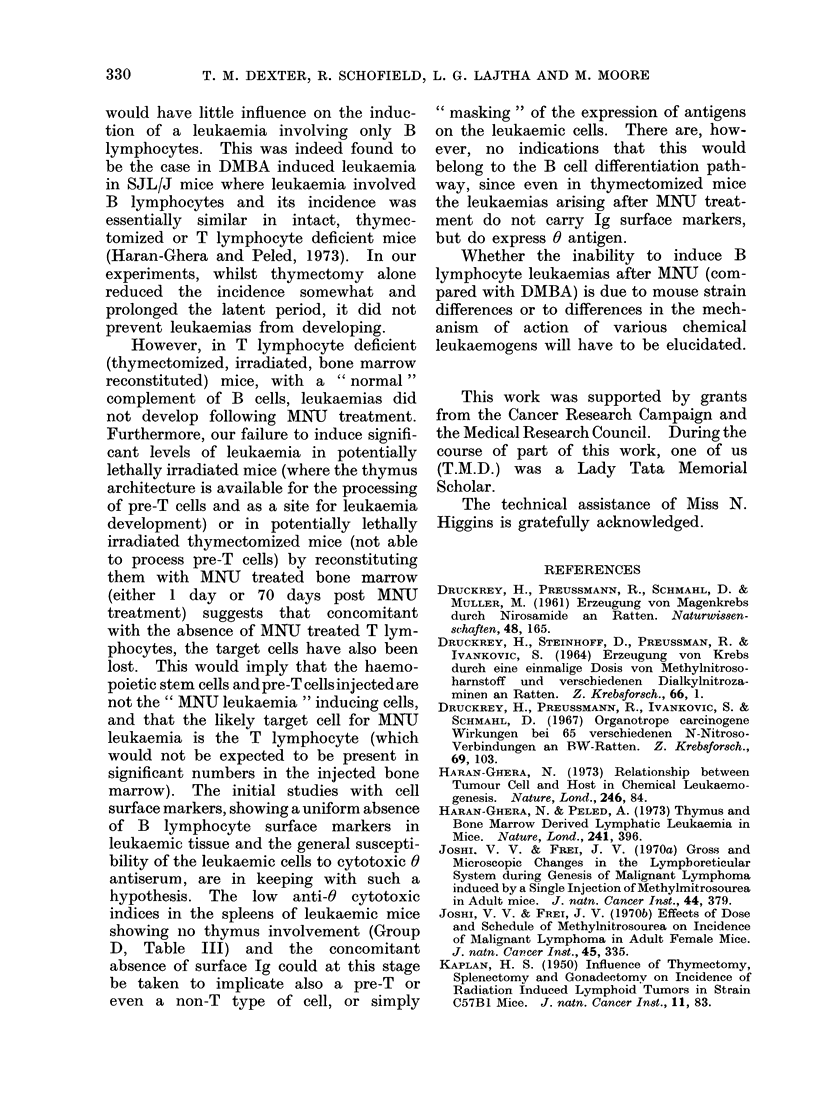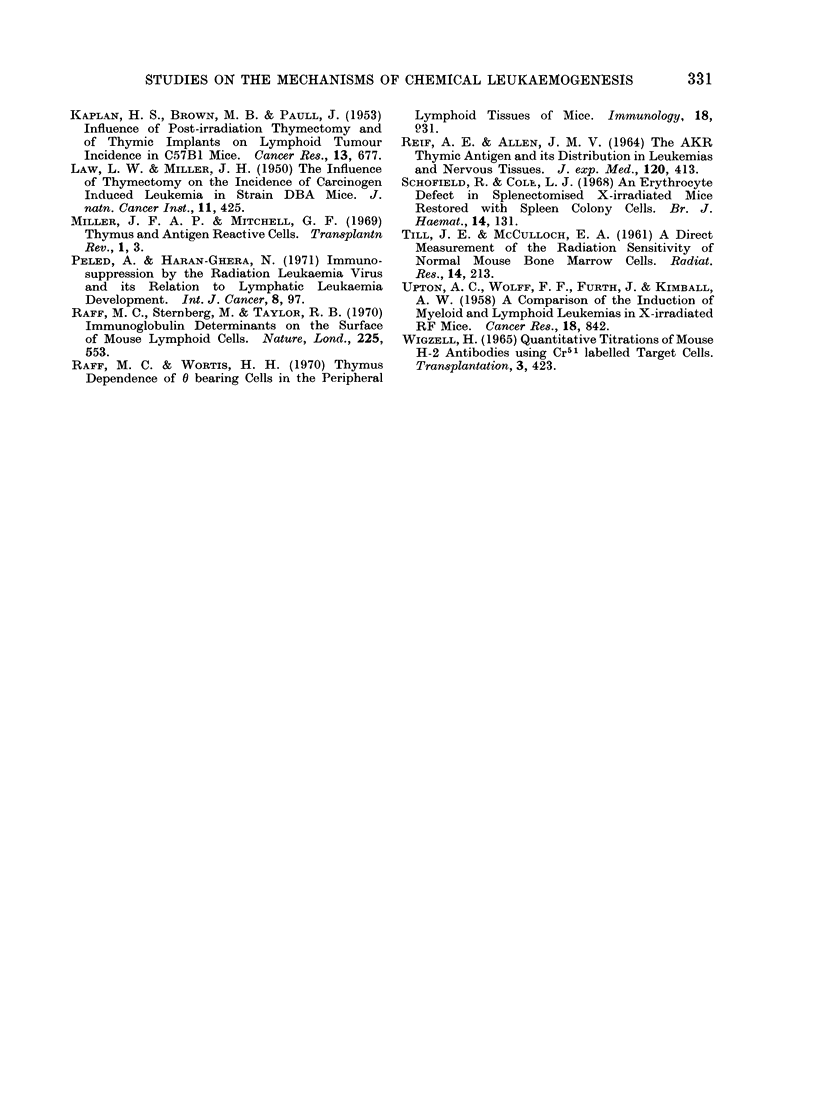# Studies on the Mechanisms of Chemical Leukaemogenesis

**DOI:** 10.1038/bjc.1974.200

**Published:** 1974-10

**Authors:** T. M. Dexter, R. Schofield, L. G. Lajtha, M. Moore

## Abstract

Following a single injection of MNU into “intact” mice, a high incidence of leukaemia (90%) is obtained, with a 50% induction time of 200 days. Immunological studies indicate that the θ antigen is expressed on the leukaemic cells. Thymectomized MNU treated mice had a 50% induction time of 500 days, and the incidence was somewhat lower. Leukaemias failed to develop in MNU treated T lymphocyte deficient animals and in lethally irradiated, or thymectomized lethally irradiated mice reconstituted with MNU treated bone marrow. It is suggested that the T lymphocytes rather than the haemopoietic stem cells or pre-T cells are the “target cells” in MNU leukaemogenesis.


*Br. J. Cancer* (1974) **30**, 325

### STUDIES ON THE MECHANISMS OF CHEMICAL LEUKAEMOGENESIS

T. M. DEXTER, R. SCHOFIELD, L. G. LAJTHA AND M. MOORE

*From the Paterson Laboratories, Christie Hospital and Holt Radium Institute, Manchester M20 9BX*

Received 4 March 1974. Accepted 14 June 1974

**Summary.—Following a single injection of MNU into " intact " mice, a high incidence of leukaemia (90%) is obtained, with a 50% induction time of 200 days. Immunological studies indicate that the $\theta$ antigen is expressed on the leukaemic cells. Thymectomized MNU treated mice had a 50% induction time of 500 days, and the incidence was somewhat lower. Leukaemias failed to develop in MNU treated T lymphocyte deficient animals and in lethally irradiated, or thymectomized lethally irradiated mice reconstituted with MNU treated bone marrow. It is suggested that the T lymphocytes rather than the haemopoietic stem cells or pre-T cells are the " target cells" in MNU leukaemogenesis.**

IN AN IMPORTANT paper recently, Haran-Ghera and Peled (1973) found that dimethylbenz(a)anthracene (DMBA) will produce specifically B lymphocyte leukaemia in several mouse strains tested, whereas radiation and virus produce T lymphocyte leukaemias. Subsequently, it was shown (Haran-Ghera, 1973) that transplant of bone marrow, 80 days after DMBA treatment, into syngeneic mice resulted in a 100% incidence of leukaemia in the recipients within 20 days. Here we describe another chemically (methylnitrosourea) induced leukaemia which shows some essential differences from the DMBA induced B lymphocyte leukaemia.

### MATERIALS AND METHODS

*Mice.*—Female BDF1 (C57B1 × DBA/2 F1) mice, 8–10 weeks old were used. Thymectomy (Kaplan, 1950) or splenectomy (Schofield and Cole, 1968) was performed when the animals were 4–6 weeks old. Experimental animals were kept 3 to a cage and supplied with food and water *ad libitum*.

*Irradiation.*—X-rays were produced by a Siemens Stabilipan x-ray machine operating at 300 kVp and 12 mA, with a half value layer of 2 mm Cu. Dose rate was 30 rad/min.

*Chemicals.*—The nitrosamide, methylnitrosourea (MNU) was chosen for the chemical induction of leukaemia. It is a highly effective carcinogen and can induce a wide variety of tumours (Druckrey *et al.*, 1961, 1964) including lymphomata in mice after a single injection (Joshi and Frei, 1970*a*, *b*). MNU was synthesized in these laboratories by Dr A. W. Craig and stored as a crystalline solid at −20°C. For experimental use MNU was dissolved in ice-cold physiological saline and the solution sterilized by passage through a millipore filter, pore-size 0·22 μm. This solution was then injected into one of the lateral tail veins of unanaesthetized mice. MNU solution was always made up freshly at a concentration of 2 mg/ml and used within 5 min of its dissolving. When the change in optical density at 230 nm was followed, in a cell maintained at 4°C by passing iced water through thermospacers, it was found that less than 10% of the compound had decomposed within the first 10 min. It has previously been shown (Druckrey *et al.*, 1967) that MNU has a short half-life *in vivo* and, in contrast with the nitrosamines, needs no enzyme activation.

*Pathology of leukaemias.*—Animals suspected of having leukaemia were killed and various tissues were taken for histological examination, including peripheral lymph nodes (superficial cervical, axillary, brachial, mesenteric and inguinal), thymus, spleen and liver, and occasionally bone marrow. Other organs were taken only if grossly involved.

In the majority of instances, the malignancy of the cells was estimated by retransplantation studies; $5 \times 10^5$–$10^7$ spleen, bone marrow or thymus cells were injected intravenously into three 8–10 week old BDF1 mice. The latent period and gross pathology of any resulting leukaemias were recorded.

*T lymphocyte deficient animals.*—These were obtained by thymectomy at 4–6 weeks of age, followed 2 weeks later by potentially lethal irradiation (850 rad x-rays) and immediate reconstitution with a graft of $10^6$ syngeneic bone marrow cells. In such animals T lymphocytes are numerically few, but normal numbers of B lymphocytes are found (Miller and Mitchell, 1969).

### RESULTS

### *Induction of leukaemia in " intact ", splenectomized, thymectomized and T lymphocyte deficient mice*

The leukaemogenic effect of a single intravenous injection of 50 mg/kg MNU is shown in Table I. Over an observation period of 500 days, no mice in the control groups developed leukaemia. MNU-treated " intact " and splenectomized animals show a similar leukaemia incidence, with 50% incidence induction times of 200 and 190 days respectively. Thymectomized mice, however, have a reduced incidence of leukaemia, with a greatly extended latent period. T lymphocyte deficient animals failed to develop leukaemia following treatment with MNU (Table I, Group B).

### *Induction of leukaemia in potentially lethally irradiated or T lymphocyte deficient mice receiving grafts of MNU treated haemopoietic cells*

In the first experiment 25 " intact " mice were treated with 50 mg/kg MNU. Twenty-four h later the animals were killed and the femoral marrow collected and diluted in Fischer's medium so that approximately 150 CFUs (spleen colony forming units: Till and McCulloch, 1961) were contained in 0·25 ml of dilution fluid. This volume of bone marrow suspension was then injected into mice irradiated with 850 rad x-rays 2 h previously. Each recipient received marrow cells from only one donor. During an observation period of 340 days, no experimental animals developed leukaemia (Table I, Group $C_1$).

In a second experiment, 25 " intact " mice were treated as above. Seventy days later the femoral and tibial marrow was removed and injected into potentially lethally irradiated (850 rad x-rays) syngeneic mice of comparable ages. Each recipient was reconstituted with bone marrow cells from only one donor, and received a graft of marrow containing approximately 4500 CFUs. Over an observation period of 180 days (*i.e.* 250 days post MNU treatment) no mice had developed leukaemia (Table I, Group $C_1$).

In a further experiment, " intact "

TABLE I.—*Induction of Leukaemia by MNU*

| Group | | Observation period (days) | Leukaemia incidence | (%) | 50% induction time (days) |
|---|---|---|---|---|---|
| A | 1 Intact controls (saline injected) | 500 | 0/40 | 0 | — |
| | 2 Thymectomized controls (saline injected) | 500 | 0/25 | 0 | — |
| B | 1 Intact (50 mg/kg MNU) | 500 | 45/50 | (90) | 200 |
| | 2 Splenectomized (50 mg/kg MNU) | 500 | 22/25 | (88) | 190 |
| | 3 Thymectomized (50 mg/kg MNU) | 600 | 17/25 | (68) | 500 |
| | 4 T lymphocyte deficient (50 mg/kg MNU) | 450 | 0/15 | (0) | — |
| C | 1 850 rad TBR* + bone marrow treated with MNU *in vivo* (24 h) | 340 | 0/25 | (0) | — |
| | (70 days) | 250 (180)† | 0/25 | (0) | — |
| | 2 Thymectomy + 850 rad TBR* + MNU treated bone marrow (150 CFUs) | 450 | 1/15 | (6·6) | — |
| D | Intact (50 mg/kg MNU + 500 rad TBR x-rays + normal bone marrow and thymus cells | 300 | 0/25 | (0) | — |

* = 850 rad x-rays, total body radiation.
† *i.e.* 180 days after grafting of bone marrow from mice treated 70 days previously with MNU.

animals were injected with 50 mg/kg MNU and the femoral bone marrow separately collected from each mouse 24 h later, as described above; 0·25 ml of bone marrow suspension (containing approximately 150 CFUs) was then injected into thymectomized animals, irradiated with 850 rad x-rays 2 h previously (the marrow from each donor being used to reconstitute only one recipient). In this group one animal out of 15 developed leukaemia (Table I, Group $C_2$) presenting as hepatomegaly.

*" Host effect " in MNU leukaemogenesis*

The possibility that the leukaemogenic activity is a secondary manifestation of a primary effect on host tissues other than those producing haemopoietic cells, was tested in the following way: " Intact " mice were treated with 50 mg/kg MNU, followed 24 h later by 500 rad x-rays (with this combination of treatments the additive cytotoxic effects are such that no animals survive without marrow grafting). Immediately after x-irradiation the animals received a graft of $2 \times 10^6$ untreated bone marrow cells (containing approximately 600 CFUs) and a subcutaneous implant (axillary region) of thymus from syngeneic mice of the same age. Out of a group of 25 mice, no animals developed leukaemia (Table ID).

*Pathology and transplantability of MNU induced leukaemia*

Histological findings are shown in Table II. The majority of leukaemias arising in MNU treated " intact " animals involved both thymus and spleen. In 18% of animals, however, *no* thymus involvement was seen. In thymectomized animals, however, spleen and liver were involved in all cases, and the mesenteric lymph node in 40% of the mice. Other lymph nodes were not involved. In all 3 groups, leukaemic cells were of the undifferentiated blast cell type. No evidence of differentiation along a particular morphological pathway was observed.

Transplantation of MNU induced leukaemias, arising in " intact " and splenectomized mice, into unirradiated adult syngeneic recipients was always successful. The latent periods between inoculation and death of the first transplant varied from 14 to 56 days, but on subsequent transfers stabilized at 10–14 days. All transplanted leukaemias were characterised by massive hepatosplenomegaly and consisted of undifferentiated blast cells. Transplantation of leukaemias arising in thymectomized mice was more difficult. Although morphologically the leukaemic cells were indistinguishable, successful transplantations were achieved only from animals presenting with simply liver and spleen involvement. In those animals

TABLE II.—*Distribution of Leukaemic Involvement in Various Tissues after a Single Injection of 50 mg/kg MNU*

| Tissue | % of animals showing involvement: Intact | Splenectomized | Thymectomized |
|---|---|---|---|
| Thymus | 82 | 84 | —* |
| Spleen | 80 | — | 100 |
| Liver | 36 | 36 | 100 |
| Lymph nodes | | | |
| Cervical | 30 | 8 | 0 |
| Axillary | 28 | 12 | 0 |
| Brachial | 24 | 12 | 0 |
| Mesenteric | 18 | 12 | 40 |
| Inguinal | 18 | 12 | 0 |

* Remants of thymus tissue were carefully looked for at time of autopsy. In only one case was thymus tissue observed and this mouse was not included in the results.

where the mesenteric lymph node was also involved, the leukaemic cells were not transplantable.

*Cell surface markers in MNU induced leukaemias*

A preliminary survey of leukaemic cells for surface markers associated with B and T lymphocytes was undertaken. Primary MNU leukaemias were tested for the presence of surface immunoglobulin (Ig) determinants, characteristic of bone marrow derived, thymus independent lymphocytes, by the direct immunofluorescence technique (Raff, Sternberg and Taylor, 1970) using fluoresceinated anti-mouse Ig (diluted 1 : 4 with PBS).

The results were compared with the fluorescent staining properties of cells derived from normal mouse lymphoid tissues. The percentage staining of lymphoid cells originating from normal spleen was 40·4 (Table III), the reactive cells revealing typical defined fluorescent " capping " of the cell surface. No stained cells were identified in normal thymocyte preparations, and insignificant numbers in leukaemic thymus preparations (Table III, Groups A, B, C). By contrast, the degree of staining of splenic lymphocytes from leukaemias arising in " intact " mice depended upon the extent to which leukaemic disease involved the organ. Thus, in those cases where the thymus and spleen were grossly involved (gross enlargement confirmed by microscopical examination) the proportion of Ig bearing cells in the spleen was 3·0% (Group A). By contrast, in one mouse where the spleen showed only a modest degree of leukaemic involvement (microscopical examination), the number of Ig bearing cells was intermediate between that of the grossly involved and disease-free organs (Group B). In those cases, however, where the leukaemia was microscopically confined to the thymus the number of Ig bearing cells (46·7%) fell within the normal range (Group C). In thymectomized control mice (assayed 450 days post thymectomy) the proportion of Ig staining cells in spleen was increased to 53%. However, in 2 leukaemias arising in thymectomized mice, the proportion of Ig bearing cells was less than 1%. These data indicate that MNU leukaemias arising in " intact " or thymectomized mice are not associated with B lymphocytes.

TABLE III.—*Expression of θ Antigen and Ig Determinants on the Cell Surface of Normal Mouse Lymphoid Tissues and MNU Induced Leukaemias*

| Group | Anti-θ cytotoxicity indices: Spleen Mean (range) | Anti-θ cytotoxicity indices: Thymus Mean (range) | Ig bearing cells: Spleen Mean (range) | Ig bearing cells: Thymus Mean (range) |
|---|---|---|---|---|
| " Intact " control | 27·3 (25·9–29·0) | 87·2 (84–90) | 40·4 (35·6–44·2) | 0 |
| " Intact " leukaemias | | | | |
| A. (6 mice) | 59·4 (46–73) | 73·5 (73·5–73·6) | 3·0 (0·1–13·7) | 0·2 (0–0·6) |
| B. (1 mouse) | ND | ND | 16·8 | 1·1 |
| C. (3 mice) | ND | 53·3 (48–58·3) | 46·7 (1 mouse) | 0·1 (0–0·1) |
| D. (5 mice) | 9·3 (2·2–13·2) | ND | 0·7 (0·1–3·1) | ND |
| Thymectomy control | 0† | — | 53·2 (52·5–53·8) | — |
| " Thymectomy " leukaemias | 53·1 (1 mouse) | — | 0·9 (0·8–1·0) (2 mice) | — |

† 450 days post-thymectomy; ND = not determined.
A. Spleen and thymus both showing gross leukaemic involvement.
B. Thymus grossly leukaemic. Spleen marginally infiltrated (microscopic examination).
C. Thymus only, showing leukaemic involvement. Spleen " normal ", *i.e.* no leukaemic infiltration.
D. Spleen involved, no thymus involvement.

The primary MNU induced leukaemias were tested also for the presence of $\theta$ antigen in a cytotoxicity assay based on release of chromium 51 (Wigzell, 1965) using AKR anti-$\theta$C3H serum prepared by the method of Reif and Allen (1964) and guinea-pig serum absorbed with agarose as a source of complement. All tests were performed in quadruplicate and the average number of counts released per tube with $\theta$ antiserum, normal mouse serum and with the detergent triton, was calculated (Raff and Wortis, 1970) and a cytotoxic index determined for each cell type according to the formula:

$$\frac{\text{counts released (CR) with antiserum} - \text{CR with NMS}}{\text{CR with triton} - \text{CR with NMS}} \times 100$$

The cytotoxic indices for 6 mice (Table III, Group A) where leukaemic disease involved the thymus and the spleen ranged from 73·5% in the thymus to 59·4% in the spleen. Whilst the indices for leukaemic thymocytes were somewhat less than that obtained for normal thymus preparations, the indices for leukaemic spleen cells were significantly higher, indicating that the leukaemias express $\theta$ antigen. In leukaemias showing only thymus involvement (Group C) a fairly high cytotoxic index is maintained for the leukaemic thymocytes. In those mice where the leukaemia involved the spleen, and *not* the thymus, uniformly low anti-$\theta$ cytotoxic indices were found for leukaemic spleen cells. This occurs concomitantly with a virtual lack of Ig bearing cells (Group D). In control thymectomized mice, spleen cells were $\theta$ antigen negative whilst, in an isolated example, splenic lymphocytes from a leukaemic thymectomized mouse were $\theta$ antigen positive, indicating here that the leukaemic cells were T lymphocytes.

### DISCUSSION

The high incidence of leukaemia seen in " intact " mice after MNU is comparable with results published by other authors (Joshi and Frei, 1970*a*, *b*), who observed a 72% incidence of malignant lymphoma in CFW/D mice after a single i.p. injection of MNU. In all these experiments a majority of leukaemias showed involvement of the thymus and infiltration of the spleen.

Although the spleen is generally involved in the presentation of the leukaemia, splenectomy had little effect upon the incidence or induction time of MNU induced leukaemias, similar to methylcholanthrene induced leukaemia in DBA mice (Law and Miller, 1950) and x-ray induced lymphoid leukaemia in RF mice (Upton *et al.*, 1958). This may mean that splenectomy does not permanently affect the number of " target cells " available for leukaemic transformation.

Leukaemias arising in thymectomized MNU treated mice seem distinct from those appearing in " intact " or splenectomized mice in that they have a prolonged induction time and are characterized by a high number of animals presenting with mesenteric lymph node involvement. The reported effect of thymectomy on the incidence of lymphoid leukaemia is variable, in some cases causing a pronounced reduction in the incidence of leukaemia (Kaplan, Brown and Paull, 1953; Law and Miller, 1950; Peled and Haran-Ghera, 1971), in others having little effect either on the induction times or the incidence of leukaemia (Haran-Ghera and Peled, 1973). The variable effect of thymectomy upon the incidence of leukaemia produced in mice by different treatments may depend upon " target " specificity of the leukaemogen. Adult thymectomy prevents further processing of pre-T cells to T cells and leads to an impairment of cell mediated immunity in old age. If the initial target cell for leukaemogenesis were the pre-T (or an earlier precursor cell) which had subsequently to be processed by the thymus before leukaemic change became apparent, then thymectomy would be expected to have an inhibitory effect on leukaemia development. However, it would have little influence on the induction of a leukaemia involving only B lymphocytes. This was indeed found to be the case in DMBA induced leukaemia in SJL/J mice where leukaemia involved B lymphocytes and its incidence was essentially similar in intact, thymectomized or T lymphocyte deficient mice (Haran-Ghera and Peled, 1973). In our experiments, whilst thymectomy alone reduced the incidence somewhat and prolonged the latent period, it did not prevent leukaemias from developing.

However, in T lymphocyte deficient (thymectomized, irradiated, bone marrow reconstituted) mice, with a " normal " complement of B cells, leukaemias did not develop following MNU treatment. Furthermore, our failure to induce significant levels of leukaemia in potentially lethally irradiated mice (where the thymus architecture is available for the processing of pre-T cells and as a site for leukaemia development) or in potentially lethally irradiated thymectomized mice (not able to process pre-T cells) by reconstituting them with MNU treated bone marrow (either 1 day or 70 days post MNU treatment) suggests that concomitant with the absence of MNU treated T lymphocytes, the target cells have also been lost. This would imply that the haemopoietic stem cells and pre-T cells injected are not the " MNU leukaemia " inducing cells, and that the likely target cell for MNU leukaemia is the T lymphocyte (which would not be expected to be present in significant numbers in the injected bone marrow). The initial studies with cell surface markers, showing a uniform absence of B lymphocyte surface markers in leukaemic tissue and the general susceptibility of the leukaemic cells to cytotoxic $\theta$ antiserum, are in keeping with such a hypothesis. The low anti-$\theta$ cytotoxic indices in the spleens of leukaemic mice showing no thymus involvement (Group D, Table III) and the concomitant absence of surface Ig could at this stage be taken to implicate also a pre-T or even a non-T type of cell, or simply " masking " of the expression of antigens on the leukaemic cells. There are, however, no indications that this would belong to the B cell differentiation pathway, since even in thymectomized mice the leukaemias arising after MNU treatment do not carry Ig surface markers, but do express $\theta$ antigen.

Whether the inability to induce B lymphocyte leukaemias after MNU (compared with DMBA) is due to mouse strain differences or to differences in the mechanism of action of various chemical leukaemogens will have to be elucidated.

This work was supported by grants from the Cancer Research Campaign and the Medical Research Council. During the course of part of this work, one of us (T.M.D.) was a Lady Tata Memorial Scholar.

The technical assistance of Miss N. Higgins is gratefully acknowledged.

### REFERENCES

Druckrey, H., Preussmann, R., Schmahl, D. & Muller, M. (1961) Erzeugung von Magenkrebs durch Nirosamide an Ratten. *Naturwissenschaften*, **48**, 165.

Druckrey, H., Steinhoff, D., Preussman, R. & Ivankovic, S. (1964) Erzeugung von Krebs durch eine einmalige Dosis von Methylnitrosoharnstoff und verschiedenen Dialkylnitrozaminen an Ratten. *Z. Krebsforsch.*, **66**, 1.

Druckrey, H., Preussmann, R., Ivankovic, S. & Schmahl, D. (1967) Organotrope carcinogene Wirkungen bei 65 verschiedenen N-Nitroso-Verbindungen an BW-Ratten. *Z. Krebsforsch.*, **69**, 103.

Haran-Ghera, N. (1973) Relationship between Tumour Cell and Host in Chemical Leukaemogenesis. *Nature, Lond.*, **246**, 84.

Haran-Ghera, N. & Peled, A. (1973) Thymus and Bone Marrow Derived Lymphatic Leukaemia in Mice. *Nature, Lond.*, **241**, 396.

Joshi. V. V. & Frei, J. V. (1970*a*) Gross and Microscopic Changes in the Lymphoreticular System during Genesis of Malignant Lymphoma induced by a Single Injection of Methylmitrosourea in Adult mice. *J. natn. Cancer Inst.*, **44**, 379.

Joshi, V. V. & Frei, J. V. (1970*b*) Effects of Dose and Schedule of Methylnitrosourea on Incidence of Malignant Lymphoma in Adult Female Mice. *J. natn. Cancer Inst.*, **45**, 335.

Kaplan, H. S. (1950) Influence of Thymectomy, Splenectomy and Gonadectomy on Incidence of Radiation Induced Lymphoid Tumors in Strain C57Bl Mice. *J. natn. Cancer Inst.*, **11**, 83.

KAPLAN, H. S., BROWN, M. B. & PAULL, J. (1953) Influence of Post-irradiation Thymectomy and of Thymic Implants on Lymphoid Tumour Incidence in C57Bl Mice. *Cancer Res.*, **13**, 677.

LAW, L. W. & MILLER, J. H. (1950) The Influence of Thymectomy on the Incidence of Carcinogen Induced Leukemia in Strain DBA Mice. *J. natn. Cancer Inst.*, **11**, 425.

MILLER, J. F. A. P. & MITCHELL, G. F. (1969) Thymus and Antigen Reactive Cells. *Transplantn Rev.*, **1**, 3.

PELED, A. & HARAN-GHERA, N. (1971) Immunosuppression by the Radiation Leukaemia Virus and its Relation to Lymphatic Leukaemia Development. *Int. J. Cancer*, **8**, 97.

RAFF, M. C., Sternberg, M. & TAYLOR, R. B. (1970) Immunoglobulin Determinants on the Surface of Mouse Lymphoid Cells. *Nature, Lond.*, **225**, 553.

RAFF, M. C. & WORTIS, H. H. (1970) Thymus Dependence of $\theta$ bearing Cells in the Peripheral Lymphoid Tissues of Mice. *Immunology*, **18**, 931.

REIF, A. E. & ALLEN, J. M. V. (1964) The AKR Thymic Antigen and its Distribution in Leukemias and Nervous Tissues. *J. exp. Med.*, **120**, 413.

SCHOFIELD, R. & COLE, L. J. (1968) An Erythrocyte Defect in Splenectomised X-irradiated Mice Restored with Spleen Colony Cells. *Br. J. Haemat.*, **14**, 131.

TILL, J. E. & MCCULLOCH, E. A. (1961) A Direct Measurement of the Radiation Sensitivity of Normal Mouse Bone Marrow Cells. *Radiat. Res.*, **14**, 213.

UPTON, A. C., WOLFF, F. F., FURTH, J. & KIMBALL, A. W. (1958) A Comparison of the Induction of Myeloid and Lymphoid Leukemias in X-irradiated RF Mice. *Cancer Res.*, **18**, 842.

WIGZELL, H. (1965) Quantitative Titrations of Mouse H-2 Antibodies using $Cr^{51}$ labelled Target Cells. *Transplantation*, **3**, 423.